# A pipeline for completing bacterial genomes using *in silico *and wet lab approaches

**DOI:** 10.1186/1471-2164-16-S3-S7

**Published:** 2015-01-29

**Authors:** Rutika Puranik, Guangri Quan, Jacob Werner, Rong Zhou, Zhaohui Xu

**Affiliations:** 1 Department of Biological Sciences, Bowling Green State University, Bowling Green, OH 43403, USA; 2 School of Software, Harbin Institute of Technology, Weihai, Shandong, 264209, China; 3 Department of Mathematics, Yuncheng University, Shanxi, 044000, China

**Keywords:** Genome assembling, GapFish, *Thermotoga*

## Abstract

**Background:**

Despite the large volume of genome sequencing data produced by next-generation sequencing technologies and the highly sophisticated software dedicated to handling these types of data, gaps are commonly found in draft genome assemblies. The existence of gaps compromises our ability to take full advantage of the genome data. This study aims to identify a practical approach for biologists to complete their own genome assemblies using commonly available tools and resources.

**Results:**

A pipeline was developed to assemble complete genomes primarily from the next generation sequencing (NGS) data. The input of the pipeline is paired-end Illumina sequence reads, and the output is a high quality complete genome sequence. The pipeline alternates the employment of computational and biological methods in seven steps. It combines the strengths of *de novo *assembly, reference-based assembly, customized programming, public databases utilization, and wet lab experimentation. The application of the pipeline is demonstrated by the completion of a bacterial genome, *Thermotoga *sp. strain RQ7, a hydrogen-producing strain.

**Conclusions:**

The developed pipeline provides an example of effective integration of computational and biological principles. It highlights the complementary roles that *in silico *and wet lab methodologies play in bioinformatical studies. The constituting principles and methods are applicable to similar studies on both prokaryotic and eukaryotic genomes.

## Background

Next-generation sequencing technologies produce massive amount of data at greatly reduced costs, making it possible to routinely sequence the genomes of various organisms. This is especially true for bacteria, whose genomes are typically less than 10 million base pairs (Mb). A standard Illumina sequencing operation can easily generate enough data to cover the genome of a bacterium more than 100 times, which often results in a near-complete genome assembly in a single attempt. In recent years, encouraging progress has been made in *de novo *sequencing for both small (for example, bacteria [1,2]) and large (for example, mammalian [3,4]) genomes. Methods for alignment and assembly [5-7] and evaluations [8,9] have also been developed. Nevertheless, no method is all-purpose, and the effectiveness of a method is often subject to constraints, such as genome size as well as the quality, length, and abundance of the reads. In addition, software and hardware environment can also play a role. As a consequence, despite the sheer volume of sequencing data and the highly sophisticated software dedicated to handling these types of data, gaps are commonly found in draft assemblies. Besides the limitations of assembling software, two other factors can lead to gaps: the nature of DNA templates and sequencing errors. Between them, the nature of DNA is more critical. For example, some regions of the genome are inherently prone to physical degradation while some others are resistant to amplification due to secondary structures. Both of these scenarios result in underrepresentation of the affected sequences in the data set, and therefore, leave gaps.

The presence of gaps often leads to errors in gene finding, annotation, and functional studies. A complete genome is thus preferred or even required in a study. One straightforward way of closing gaps is conducting wet lab experiments, that is, primer walking and Sanger sequencing. However, this approach can be prohibitive, in terms of costs. Here we report a pipeline aimed to assembling complete genomes with a combination of *in silico *and wet lab approaches. The input of the pipeline is paired-end sequence reads generated by the Illumina technology, and the output is a high quality complete genome sequence. The genome being used as an example belongs to the hyperthermophilic bacterium *Thermotoga *sp. strain RQ7, which has a circular genome about 1.8 ~ 1.9 Mb, as estimated based on its close relatives. *Thermotoga *are potential producers of biohydrogen gas [10], a type of clean, renewable fuel.

## Methods

### Polymerase chain reaction (PCR)

The genomic DNA of *T*. sp. strain RQ7 was prepared by three rounds of phenol extraction [11]. Primers used in all PCR reactions were designed with the assistance of Primer3 [12,13] and Clone Manger[14]. The sequences of PCR products were determined by Sanger sequencing. All wet lab experiments were performed according to standard procedures.

### Pipeline of scaffold assembling and gap closure

**Figure 1:**
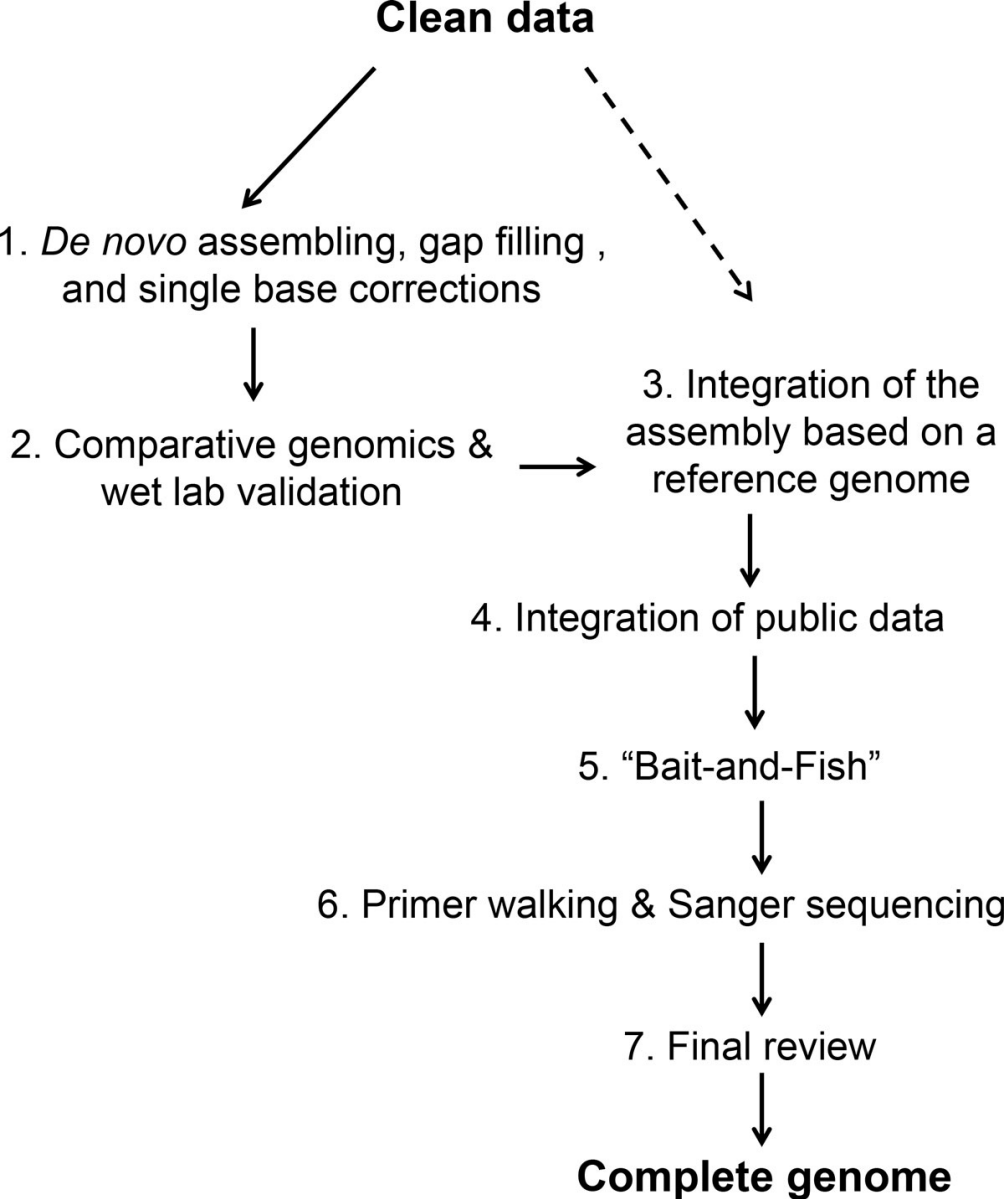
**The pipeline of genome assembling and gap closure**. Clean data were subject to *de novo *assembling, initial gap filling, and single base corrections with SOAPdenovo and SOAPaligner; the resulting assembly was used for comparative genomics studies and also provided guidance for wet lab validations. Meanwhile, the clean data were assembled separately based on a reference genome using CLC Genomics Workbench. This second assembly was integrated into the first one to yield a hybrid assembly, which was then updated with public data, GapFish results, and Sanger sequencing data until the genome sequence was complete.

A modular pipeline consisting of seven components (Figure 1) was developed to meet our assembling needs:

Step 1. *De novo *assembling with the SOAP package. Three sequencing libraries, with inserts in sizes of 500 bp, 2000 bp, and 5000 bp, were prepared from the genomic DNA of *T*. sp. strain RQ7. Paired-end sequencing was performed with each library, which generated reads of 90 bp for the 500 bp library and of 49 bp for the other two libraries. A total of 400 Mb clean data were collected and were assembled by SOAPdenovo [3] with K = 33. K value determines the quality of assembly, as measured by the size of the assembly and the number of "N"s it has. Larger K values generate longer contigs, but require deeper sequencing depth and longer reads [3,5,6]. When assembling microbial genomes from Illumina data, K is often set between 25 and 40. In this work the Kmer was empirically set at 33 for best results. After the assembling, an initial gap filling and a single base correction were undertaken with SOAPaligner [15]. This part of the work was done by BGI Americas (Cambridge, MA) under a service agreement.

Step 2. Comparative genomics and wet lab validation. A comparative genomics study was performed to evaluate the above assembly and to identify the closest relatives of the *T*. sp. strain RQ7. PCR and Sanger sequencing were conducted to validate the analyses.

Step 3. Integration of the assembly based on a reference genome. An independent assembling effort was taken using the commercial software package CLC Genomics Workbench [16], based on the complete genome sequence of a close relative, which had been identified in Step 2. This assembly was combined with the one obtained in Step 1 to give rise to a hybrid assembly, which was further updated in later steps.

Step 4. Integration of public data. Public databases were searched for sequences belonging to *T*. sp. strain RQ7. The retrieved sequences were used to validate the assembly of Step 3 and to fill in the gaps in the hybrid assembly.

**Figure 2:**
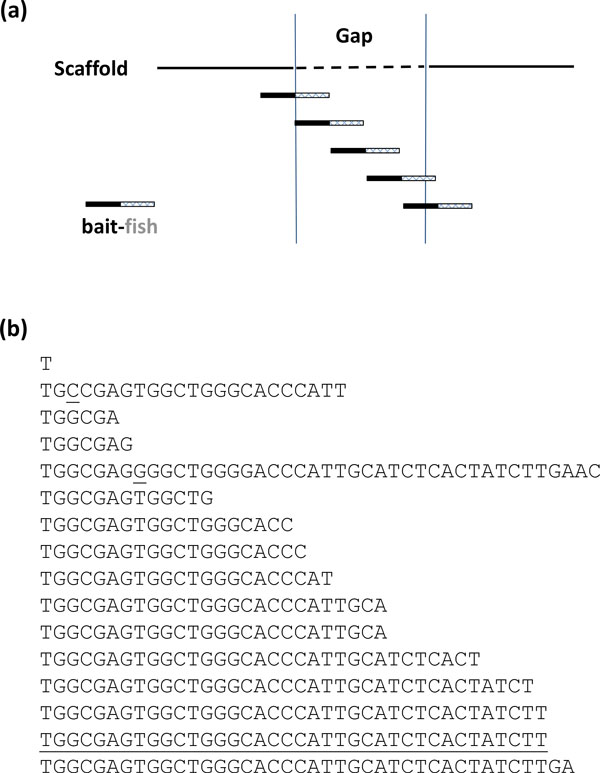
**Schematic overview of the GapFish algorithm**. **(a) **A segment upstream of a gap will be used as the "bait" to search against all Illumina reads that are 90 nt long. If the "bait" is found in a read, GapFish will excise the fragment adjacent to the "bait" at the 3' direction and return the result (the "fish") to the console. At the end of each search, all identified fragments will be sorted and save into a text file. **(b) **An example output of GapFish when searching with "bait" = 'GAGGCTCCTCAGGCGGTTGTGGAGGGCAATCCCAGAAACTCCG' (total 43 nt). Sequencing errors are apparent in the results, such as the 3**^rd ^**position (G -> C) in the second line and the 8**^th ^**position (T -> G) in the fifth line (both are underlined). This type of errors could have led to the collapse of the assembling effort of SOAPdenovo, leaving a gap behind. For solving this type of complications, GapFish-assisted human interventions have proven to be necessary. The sequence second to the last one (also underlined) will be used as the "bait" for the next round of search.

Step 5. "Bait-and-fish". This step intends to close the remaining gaps with GapFish (Figure 2), an in-house program written in Python. GapFish adopts the "bait-and-fish" (or "seed-and-extend") scheme. The input of the program is a sequence found upstream of the gap, which is used as the "bait". The output is a sorted list of all sequences (the "fish") located in the same reads with the "bait" but immediately downstream of them. The list is evaluated for consensus sequences, and new "bait" is formulated, which is usually the second or third longest "fish". The process is iterated until the gap is filled (Figure 2). A gap is considered filled when the new sequence is mapped to the other side of the gap and the total length of the new sequences is about the size of the gap. When there are competing consensus sequences, the search process will be branched out until a fished out sequence matches to the downstream of the original gap. This process requires human intervention after each search cycle. Depending on the context of each gap, the user can define the length and the location of the "bait" and specify the source data set to search within. By this stage, only the most problematic gaps remain, which demands constant monitoring and intervening from the user. In this work, we typically started from the immediate upstream of a gap. The baits were ~45 bp in length, and the source data were the two files containing 90 bp reads. The expected size of the fished out sequences were ~45 bp or less, depending on the position of the bait in the matched read. The further the "bait" toward the 5' of the read, the longer the "fish" will be. GapFish can be downloaded from http://personal.bgsu.edu/~zxu/.

Step 6. Closing the remaining gaps with primer walking and Sanger sequencing. This was to fill in the remaining gaps and to validate the results of the previous steps. Optimization of DNA preparation and PCR conditions were frequently required. As expected, this was the most costly, time-consuming, and labor-intensive step.

Step 7. Final review with GapFish. Although GapFish was originally written to fill in the difficult gaps, we found it was also handy in the final review stage. It was used to double check the overall assembly, especially the sites surrounding the original gaps. Errors, introduced when replacing the "N"s with specified sequences, were corrected. GapFish was also useful in circularizing the genome. It was used to remove overlapping region at the ends of the linear assembly. In case the ends of the linear assembly do not meet, GapFish can be used to fill in the gap between the ends.

## Results

### Completing the genome

Illumina sequencing generated 400 Mb of clean data, which was expected to cover the genome of *T*. sp. strain RQ7 more than 200 times. After assembling with SOAPdenovo and gap-filling with SOAPaligner (Step 1), a scaffold of 1,822,593 bp (including 14,240 "N"s) was available (Table 1), which corresponds to ~98% of the complete genome. The scaffold had 27 gaps in the range of 1 bp to 3.2 kb, represented by strings of "N"s. Each of these gaps seemed to be small enough to be closed by a single PCR reaction; thus these gaps were referred to as minigaps.

**Table 1:** Comparison of the assemblies generated by different methods.

Methods	Scaffold size (including 'N's)	# of 'N's	Total nt assembled	Coverage*	# of gaps	Max gap
SOAP package	1,822,593	14,240	1,808,353	97.7%	28	~36 kb

CLC package	1,884,513	201,850	1,682,663	90.9%	380	~21 kb

This pipeline	1,851,618	0	1,851,618	100%	0	0

*Coverage was calculated by comparing the total number of nucleotides (nt) assembled (without "N"s) to the size of the complete genome of *T*. sp. strain RQ7.

A comparative genomics study (Step 2) was conducted by examining the assembly against all publicly available *Thermotoga *genomes. It was found that the genome of *T*. sp. strain RQ7 was most similar to that of *T*. *neapolitana*, indicating that the two strains are more closely related to each other than to any other *Thermotoga *strains. This finding is in agreement with the results based on 16s rRNA analysis [17]. Therefore, the genome of *T*. *neapolitana *was used as the reference genome in later part of the study. Meanwhile, it did not escape our attention that although the genomes of *T*. sp. strain RQ7 and *T*. *neapolitana *shared a high level of synteny, small scales of insertions, deletions, and rearrangements were common. Most strikingly, a region of ~36 kb found in *T. neapolitana *was missing in the assembly of *T*. sp. strain RQ7. Further investigation indicated that this region was conserved among other *Thermotoga *genomes (Table 2). This unusually large deletion, observed only in the current assembly of *T*. sp. strain RQ7, prompted us to test the authenticity of the deletion by wet lab experiments. Five genes of the region were arbitrarily selected for amplification. PCR primers were designed based on the conserved parts of these genes, and genomic DNA from four species was tested, namely, *T. neapolitana*, *T. maritima*, *T*. sp. strain RQ2, and *T*. sp. strain RQ7. The PCR profiles and the following Sanger sequencing results revealed that all of the 5 tested genes were present in *T*. sp. strain RQ7, which means, in addition to the 27 minigaps, the current assembly contained a hidden big gap of ~36 kb.

**Table 2:** Comparison of the big gap region among different *Thermotoga *genomes.

*T. maritima*	*T*. sp. strain RQ2	*T. neapolitana*	*T*. sp. strain RQ7	Annotation
TM0968	TRQ2_1822	CTN_1608	Present	hypothetical protein

TM0969	TRQ2_1821	CTN_1607	Present	hypothetical protein

TM0970	Absent	CTN_1606	Disrupted	hypothetical protein

TM0971	TRQ2_1821	Present*	Present	hypothetical protein

TM0972	TRQ2_1820	CTN_1605	Disrupted	conserved hypothetical protein, GGDEF domain

TM0973	TRQ2_1819	CTN_1604	Present	hypothetical protein

TM0974	TRQ2_1818	CTN_1603	Present	hypothetical protein

TM0975	Absent	CTN_1602	Disrupted	hypothetical protein

TM0976	Absent	Present*	Present	hypothetical protein

TM0977	Absent	CTN_1601	Present	hypothetical protein

TM0978	TRQ2_1817	CTN_1600	Present	hypothetical protein

TM0979	TRQ2_1816	CTN_1599	Present	hypothetical protein

TM0980	TRQ2_1815	CTN_1598	Present	hypothetical protein

TM0981	TRQ2_1814	CTN_1597	Disrupted	hypothetical protein

TM0982	TRQ2_1813	CTN_1596	Present	hypothetical protein

TM0983	TRQ2_1812	CTN_1595	Disrupted	hypothetical protein

TM0984	TRQ2_1811	CTN_1594	Disrupted	hypothetical protein

TM0985	TRQ2_1810	CTN_1593	Present	hypothetical protein

TM0986	TRQ2_1809	CTN_1592 & CTN_1591	Present	hypothetical protein

TM0987	TRQ2_1808	CTN_1590	Disrupted	hypothetical protein

TM0988	TRQ2_1807	CTN_1589	Disrupted	hypothetical protein

TM0989	TRQ2_1806	CTN_1588	Present	hypothetical protein

TM0990	TRQ2_1805	CTN_1587	Disrupted	hypothetical protein

TM0991	TRQ2_1804	CTN_1586	Disrupted	hypothetical protein

TM0992	Absent	CTN_1585	Disrupted	hypothetical protein

TM0993	Absent	CTN_1584	Present	hypothetical protein

TM0994	Absent	CTN_1583	Present	hypothetical protein

TM0995	Absent	CTN_1582	Present	hypothetical protein

TM0996	TRQ2_1803	CTN_1581	Present	hypothetical protein

TM0997	TRQ2_1802	CTN_1580	Disrupted	hypothetical protein

TM0998	TRQ2_1801	CTN_1579	Present	transcriptional regulator, ArsR family

TM0999	Disrupted	Present*	Present	hypothetical protein

TM1000	Absent	CTN_1578	Present	hypothetical protein

TM1001	Absent	CTN_1577	Present	hypothetical protein

TM1002	TRQ2_1800	CTN_1576	Disrupted	hypothetical protein

TM1003	Absent	CTN_1575	Absent	hypothetical protein

TM1004	TRQ2_1800	CTN_1573	Absent	hypothetical protein

Comparative analysis of the ~36 kb big gap region among four *Thermotoga *genomes, showing the synteny and conservation. This region was missing from the initial assembly of the *T*. sp. strain RQ7 genome but was included after combining the data from the CLC assembly and the primer walking effort. The asterisks (*) indicate the presence of a homolog that is not annotated as a gene in a particular genome. Absent indicates the complete deletion of the ORF in a particular genome.

In order to reduce the gap number and/or their sizes, a commercial software package CLC Genomics Workbench was employed to re-assemble the entire set of Illumina reads of *T*. sp. strain RQ7, using *T*. *neapolitana *as the reference genome (Step 3). An assembly of 1,884,513 bp (including 201,850 "N"s) was generated, which had 380 gaps, ranging from 1 bp to 21 kb (Table 1). The scaffolds generated by the CLC package filled 12 of the 27 minigaps found in the SOAP assembly. After patching in the sequences of the 12 minigaps, we obtained a hybrid assembly of 1,823,180 bp (including 7,544 "N"s). Furthermore, 7 fragments totalling up to 12,511 bp were assigned to the big gap, which brought the total assembled nucleotides to 1,828,147 bp (Table 3).

**Table 3:** Statistics of the assembling process

	Step 1	Step 2	Step 3	Step 4	Step 5	Step 6	Step 7
**# Minigaps**	27	27	15	13	1	0	0

**Assembled nt in the big gap**	0	0	12,511	12,511	12,511	35,746	35,746

Total nt assembled*	1,808,353	1,808,353	1,828,147	1,828,363	1,832,588	1,851,716	1,851,618

*: "N"s are not counted. Assemblies in Steps 1-6 have overlapping end sequences (terminal redundancy). As a result, the assembly in Step 6 appeared to be slightly bigger than the final assembly.

Searching of GenBank (Step 4) identified 13 entries belonging to *T*. sp. strain RQ7, which were Sanger reads previously deposited by other researchers. Among them, 11 fell into the assembled regions and 2 into the unfilled minigaps. After that, GapFish (Step 5) successfully closed 12 more minigaps. The last remaining minigap, located in a highly repetitive region, could only be reliably determined by wet lab experiment. In addition, we were able to use GapFish to correct 4 locations that had been previously misassembled by either the SOAP package or the CLC package. However, the attempt to fill in the big gap with GapFish revealed that there were no additional reads in the data set that belonged to the big gap. This surprising finding suggested that sequences of this region were severely underrepresented in the original data set. Under such circumstances, we had to appeal to the wet lab approach for the remaining tasks.

Primer walking and Sanger sequencing (Step 6) were sought after to solve the last mini-gap, to validate the filling of other minigaps containing repetitive sequences, and to close the big gap. The closure of the big gap was achieved by using freshly prepared DNA template (less than 5 days) and optimizing PCR conditions. The requirement of fresh DNA templates indicated the physical instability of the region, which could have led to the underrepresentation of the corresponding sequence reads. Eventually, the total size of the big gap was determined to be 35,746 bp. This region of the *T*. sp. strain RQ7 genome was compared to the counterparts of *T. neapolitana*, *T. maritima*, and *T*. sp. strain RQ2. Most of the predicted gene products are hypothetical proteins with unknown functions (Table 2). After a final review (Step 7), the complete genome was determined to be 1,851,618 bp, with a GC content of 46.13%. The statistics of the assembling process is summarized in Table 3. To highlight the effectiveness of the pipeline, the final assembly was compared to the assemblies obtained just from the SOAP or CLC package (Table 1). Because our pipeline integrates results from both packages, it is clearly superior to either of them employed alone. By further integration of the data from other sources, including the public data, the GapFish analyses, and the wet lab experimental data, the pipeline was able to deliver a complete genome in the end. In our pipeline, the results of each step were evaluated and validated multiple times in the following steps, which ensures high quality of the final assembly. The genome of *T*. sp. strain RQ7 has been deposited to GenBank with the accession number CP007633.

### Differentially annotated genes

Because gaps in draft genomes may contain start and stop codons, closing the gaps is likely to alter the annotation results of the affected regions. Compared to the initial draft assembly of the SOAP package, the complete genome of *T*. sp. strain RQ7 recovered 42 genes that were previously missing, changed the sizes of 12 genes, and dismissed 9 false open reading frames (ORFs) (Table 4). The affected genes were predicted to take part in many essential cellular processes, such as sugar transportation and utilization, chemotaxis, RNA/DNA processing, sulfur metabolism, and transcription regulations. Missing or false annotation of these genes could lead to erroneous interpretation of the biology of the organism, which could result in great loss if follow-up work was pursued with these genes.

**Table 4:** ORFs differentially annotated in the complete genome

	# of affected ORFs	Putative functions
**Size variation**	12	ABC-type sugar transport and utilization machinery; chemotaxis protein; RNA/DNA processing

**Recovered ORFs**	42	ABC-type sugar transport and utilization machinery, transcriptional regulators, sulfur metabolism system, DNA/RNA helicases, DNA methylases

**Dismissed ORFs**	9	-

## Discussion

This work distinguishes itself from similar studies [18,19] from the aspect of multi-phase interactions between computational and biological approaches (Figure 1). The pipeline started from a *de novo *assembly of paired-end reads, followed by a comparative genomics analysis and wet lab verification, which led to the identification of a reference genome as well as the discovery of a hidden big gap. The genome was then re-assembled based on the reference genome, providing additional contigs. Public databases were then utilized to reduce the number of gaps, prior to another round of customized gap closure effort, using GapFish. When *in silico *options were exhausted, wet lab methods were introduced again; by then, the number of required lab work had been reduced to the minimum. The pipeline ended with an *in silico *final review.

The robustness of the pipeline relies upon the intimate interaction of *in silico *and wet lab approaches from an early stage. Rich biological information deduced from the previous step of computation can be used to guide the next stage of work, either *in silico *or in a wet lab setting. Because *in silico *methods are more efficient and less expensive, we should take full advantage of their benefits. In this work, software products from various sources were utilized, including the free ware SOAPdenovo and SOAPaligner, the commercial package CLC Genomics Workbench, and the in-house program GapFish. They accomplished complementary tasks, and each played a critical role at different stages. On the other hand, technologies have their shortcomings. Computational results should always be carefully evaluated in a biological context and be validated with wet lab experiments.

The pipeline is intrinsically flexible to allow customization of the modules. For instance, one can simply skip Step 4 if there are no public data available for the target genome. In addition, it is worthwhile to point out that the reference genome used in Step 3 refers to the most related genome sequence currently available. The reference genome would be most helpful if it is from the same species as the target genome, but it could still provide useful regional assemblies if they just share the genus (as *T*. sp. strain RQ7 and *T. neapolitana *do). Bypassing this step is also acceptable, although it will shift the workload to the later steps.

## Conclusions

In this study, we developed a genome assembling pipeline using commonly available tools and resources. It stresses the intimate, multi-phase interactions between *in silico *and wet lab approaches. The application of the pipeline was established via the delivery of the complete genome sequence of *T*. sp. strain RQ7. The constituting principles and methods are applicable to a variety of similar studies with both prokaryotes and eukaryotes.

## Competing interests

The authors declare that they have no competing interests.

## Authors' contributions

RP conducted all PCR experiments and performed most part of the data analysis. JW, GQ, RZ, and ZX participated in data analysis. RP, GQ, and RZ also contributed to drafting the manuscript. ZX conceived and coordinated the study, wrote the GapFish software, and drafted the manuscript. All authors read and approved the final manuscript.

## Declarations

Funding for publication of the article was provided by the corresponding author's institution.

This article has been published as part of *BMC Genomics *Volume 16 Supplement 3, 2015: Selected articles from the 10th International Symposium on Bioinformatics Research and Applications (ISBRA-14): Genomics. The full contents of the supplement are available online at http://www.biomedcentral.com/bmcgenomics/supplements/16/S3.
